# Remote Assessment of Parkinson Disease Using Deep Learning on Structured Mouse-Trace Data From Suspected Cases: Machine-Learning Pilot Feasibility Study

**DOI:** 10.2196/81368

**Published:** 2026-06-26

**Authors:** Md Rahat Shahriar Zawad, Zerin Nasrin Tumpa, Lydia Sollis, Shubham Parab, Peter Washington

**Affiliations:** 1 Department of Electrical and Computer Engineering, Rice University, Houston, TX, United States; 2 Department of Information and Computer Sciences, University of Hawaiʻi at Mānoa, Honolulu, HI, United States; 3 Department of Computer Science, New York University, New York, NY, United States; 4 Department of Medicine, Division of Clinical Informatics and Digital Transformation, University of California, San Francisco, 10 Koret Way, San Francisco, CA, 94143, United States, 1 4153532067

**Keywords:** Parkinson disease, feasibility study, remote screening, mouse-trace data, digital diagnostics

## Abstract

**Background:**

Parkinson disease (PD) is a pervasive neurodegenerative disorder globally, largely characterized by motor symptoms. Most existing artificial intelligence models for PD detection are trained on participants in well-resourced settings with confirmed clinical diagnoses. However, specialist-confirmed labels are often infeasible in low-resource settings.

**Objective:**

We developed a web platform for structured mouse data collection through pattern tracing tests. We sought to assess the feasibility of leveraging data from a community-recruited sample of participants with suspected but undiagnosed PD to train artificial intelligence models that achieve respectable performance in predicting diagnosed PD. We tested whether using weaker diagnostic labels that may be more feasible to collect in community or global health settings, where access to professional neurologists is sparse or nonexistent, can lead to models that learn predictive signals that are diagnostically useful.

**Methods:**

261 participants (73 self-reported PD, 155 non-PD, and 33 suspected PD) were recruited from community organizations in Hawaii and completed 3 pattern tracing tasks on our custom web assessment: straight line, sine wave, and spiral wave. During each task, cursor positions, screen dimensions, and an in-target boolean flag were recorded. From these data, we engineered features and generated mouse trace images. We built 3 categories of classifiers: (1) a feed-forward neural network using engineered features, (2) fine-tuned computer vision deep learning models, and (3) multimodal models concatenating a feed-forward neural network with computer vision models. Performance was evaluated using 1 primary experiment and 2 secondary analyses. The primary experiment involved training on suspected PD versus non-PD and testing on self-reported PD versus non-PD. A secondary analysis evaluated the reverse direction by training on participants with self-reported PD and without PD and then testing on participants with suspected PD versus participants without PD. Additionally, a cross-validation analysis was conducted using participants with self-reported PD versus those without PD with 5-fold cross-validation to establish baseline performance under well-defined diagnostic labels.

**Results:**

The best-performing models included a multimodal Vision Transformer in the primary experiment (*F*_1_: mean 0.7619, SD 0.0535), a multimodal ResNet-50 in the secondary analysis (*F*_1_: mean 0.9353, SD 0.0334), and an image-based DenseNet-201 in the cross-validation analysis (*F*_1_: mean 0.9027, SD 0.0332). Training on patients with suspected PD yielded meaningful performance in predicting self-reported PD, supporting the feasibility of using lower-specificity labels for model development.

**Conclusions:**

This pilot feasibility study suggests that remotely collected mouse-tracing data can support PD screening models under data labeling conditions of low diagnostic specificity: models trained on suspected PD from a community sample may learn signals that can transfer to predicting actual PD. Future work may consider pretraining using weaker labels and then fine-tuning on stronger clinical labels.

## Introduction

Parkinson disease (PD) is a neurodegenerative disorder that significantly impacts the central nervous system. Major symptoms include tremors, bradykinesia, muscle rigidity, and postural instability [1,2], which progressively worsen over time, leading to difficulties in performing routine tasks such as typing and using a mouse. The progression of these symptoms significantly affects quality of life, making early and accurate diagnosis crucial to enable early intervention [3]. PD is the second most common neurodegenerative disease after Alzheimer disease, affecting approximately 10 million people globally. In the United States, around 1 million individuals are diagnosed with PD, with an annual increase of about 90,000 new cases. This number is projected to rise to 1.2 million by 2030 [3,4]. Currently, there is no definitive biomarker for PD, and diagnosis is primarily based on clinical symptoms and neuropsychological tests such as the Mini-Mental State Examination and the Unified Parkinson Disease Rating Scale [5-7]. These tests involve questionnaires and subjective evaluations by clinicians, which can lead to significant biases and potential misdiagnoses [6]. This is particularly problematic, as PD symptoms often overlap with those of other age-related conditions and drug-induced Parkinsonism [8-10]. Additionally, PD is characterized by the degeneration of dopamine-producing neurons in the brain, and by the time motor symptoms become apparent, approximately 30% to 50% of these neurons have already deteriorated [11]. Therefore, improving approaches for more accessible classification of PD remains an important goal for effective management and treatment.

Previous digital health research on PD classification has included the analysis of hand and finger movements, keystroke dynamics, speech, handwriting, drawing tests, gait analysis, and sensor data from accelerometers and gyroscopes [12-27]. The use of sensors placed on lower limbs, wearable sensors supported by video recordings, and sensing coils or paper-based pads has proven effective in classifying PD and detecting tremors [16-19,22,25-30]. However, these methods often require controlled laboratory settings and specialized devices, limiting their broader applicability. Self-administered methods, such as keyboard interactions, keystroke dynamics, and smartphone screen interactions, have also been explored [14,15,21], but they may introduce biases, particularly against individuals with slower typing speeds. Mobile apps for data collection, symptom monitoring, and treatment management have demonstrated utility in tracking activities such as finger-tapping speed, gait, and motor performance [31-33], though they may pose challenges for older adults unfamiliar with smartphone technology. Moreover, video-based evaluations of finger tapping, facial expression, hand movement, and gait analysis have shown strong performance in PD classification [34-37]. Multimodal approaches combining different data types, such as speech, gait, and upper limb movement data, have also demonstrated potential in classifying patients with PD [38,39].

Despite the prior success of digital PD biomarkers, these tools have traditionally been trained using data collected from well-resourced settings. Transferring such models for use in lower-resourced settings often requires training or fine-tuning the models on data from those settings. However, it is often only feasible to obtain lower-specificity, community-collected data labels in these settings. We sought to understand how well labels of suspected PD transfer to predicting diagnosed PD.

To support this objective, we structured our evaluation around 1 primary experiment and 2 secondary analyses, each reflecting different levels of diagnostic certainty and real-world deployment scenarios. The primary experiment involved training models on participants with suspected PD and without PD and evaluating them on patients with self-reported PD and without PD. This experiment directly addresses our central research question by assessing whether models trained using weaker, low-specificity, community-recruited labels can be diagnostically useful. We also conducted a secondary analysis examining the reverse direction, in which models were trained on participants with self-reported PD and without PD and tested on individuals with suspected PD and without PD. This analysis was included to assess whether the learned signal is bidirectional. Additionally, we conducted a cross-validation analysis using only participants with self-reported PD and without PD to establish baseline performance under well-defined diagnostic labels. Importantly, the suspected PD group in this study is based solely on the self-report and does not represent clinically confirmed PD. Instead, it reflects a heterogeneous population with uncertain diagnostic status, which we use to model real-world diagnostic ambiguity rather than validated prodromal diseases. Accordingly, this study is positioned as a feasibility investigation to determine whether meaningful PD-related signals can be learned under conditions of diagnostic uncertainty.

## Methods

### Overview

**Figure 1.**
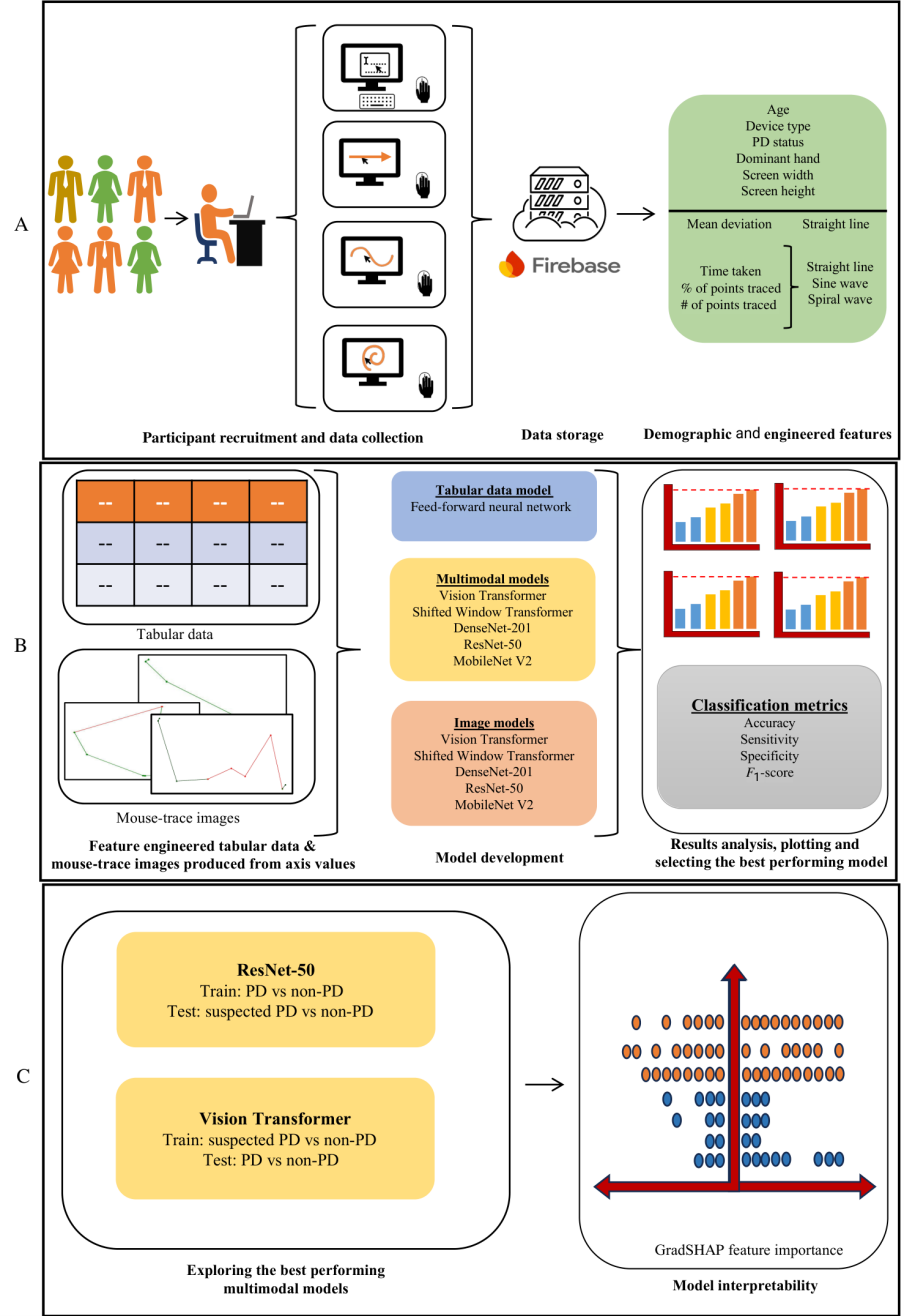
The study workflow from data collection to model evaluation and interpretability analysis. (A) Participants completed the data collection process remotely on the website. (B) Engineered features and mouse-trace images were fed into 3 classes of models. The results were analyzed and visualized to identify the best performing models. (C) The best multimodal models (multimodal ResNet-50 and multimodal ViT) were analyzed for interpretability using GradSHAP (Gradient Shapley Additive Explanations) feature importance. DenseNet-201: Densely Connected Convolutional Network-201; MobileNet V2: Mobile Network v2; PD: Parkinson disease; ResNet-50: Residual Network-50; ViT: Vision Transformer.

We created a website to collect structured mouse movement data by having participants complete a series of mouse-tracing tests remotely. We trained a series of deep-learning models using various features derived from the mouse traces. Figure 1 outlines the workflow from data collection to model evaluation and interpretability analysis.

### Participant Recruitment and Data Collection

We recruited participants for this study through both online methods (email and social media posts) and offline methods (community meetings and conventions in Hawaii). We collaborated with the Hawaii Parkinson’s Association and Beyond Rehab to post flyers and advertise the study to various PD emailing lists. Additionally, we established a recruitment booth at the 2024 Hawaii Parkinson’s Association Symposium and the 2024 Hawaii Annual Parkinson’s Run, where we provided potential participants with flyers describing how to complete the study.

**Figure 2.**
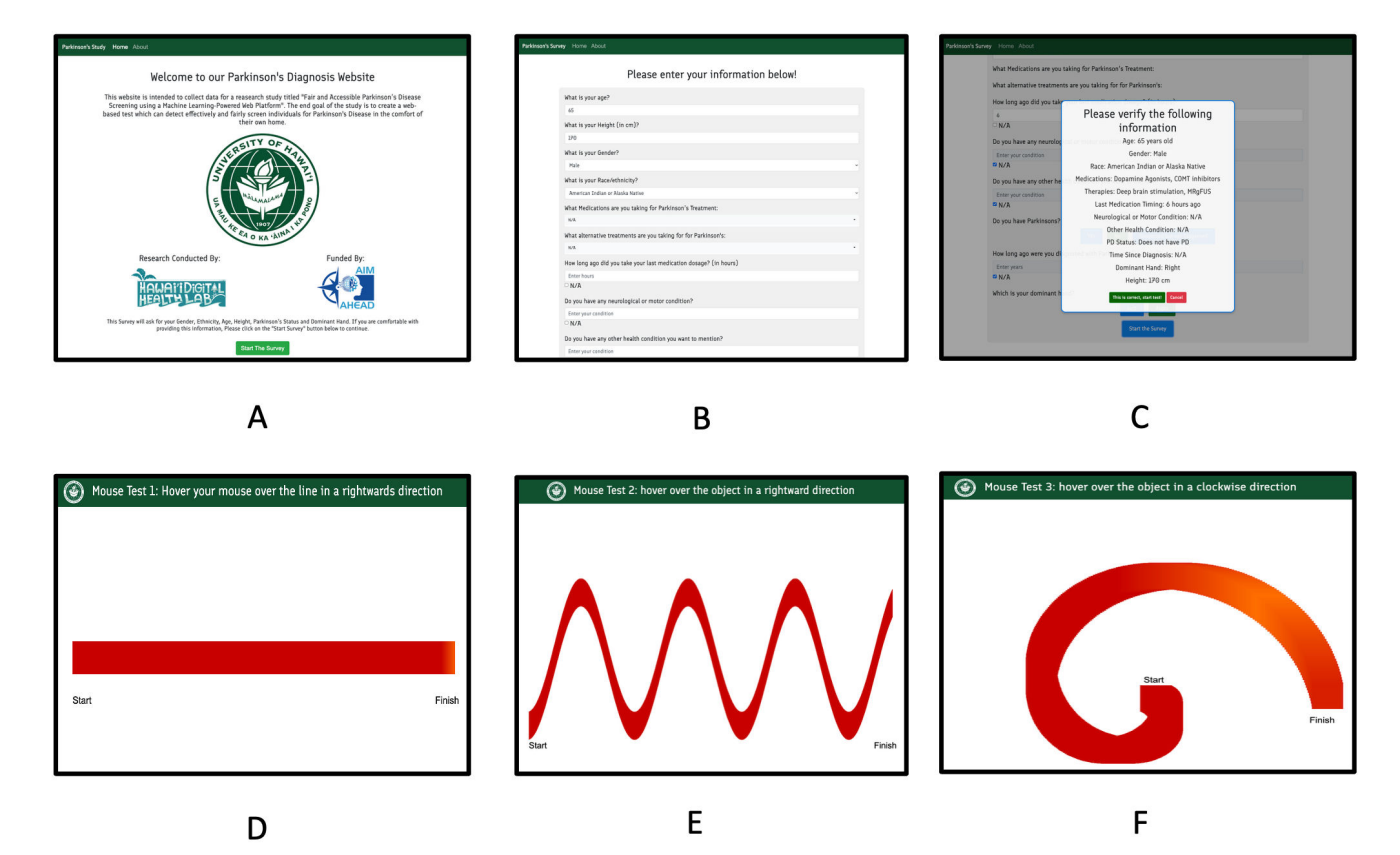
Pages of the data collection website: (A) Introduction page of the website to inform participants about the study; (B) Participants were asked to provide information about themselves; (C) Participants were asked to confirm their information to prevent mistakes; (D) Participants were asked to trace a straight line; (E) Participants were asked to trace a sine wave; (F) Participants were asked to trace a spiral wave.

We collected data via a web application that we developed [40], illustrated in Figure 2. Participants provided demographic and disease-related information, including age and dominant hand. Due to the infeasibility of obtaining official diagnostic documents for all participants under the constraints of the pilot grant that funded this project, we asked participants to self-report their PD status.

Notably, we provided an option to select “suspected PD” for participants who think that they might have PD but are yet to receive a formal diagnosis, as nonmotor symptoms and subtle, nonspecific motor signs may appear before a formal PD diagnosis, which is often referred to as the prodromal phase of PD [41]. For example, in the Rotterdam Study, it was found that among individuals who developed PD after a mean follow-up of 5.8 years, 71.8% had reported at least one early subjective motor symptom [42]. For patients below 50 years, it typically takes over 2 years to receive an official PD diagnosis [43]. Moreover, disparities in the health care system make it harder for people to access a diagnosis, and non-White Americans in the United States are half as likely to be diagnosed with PD [44]. Additionally, in low-income and middle-income countries (ie, Kenya), a diagnosis of PD is often followed by a period of uncertainty where, sometimes, a diagnostic decision is reversed [45].

However, participants who selected “suspected PD” did so based on their personal concerns or perceived symptoms and were not required to report or demonstrate clinically observable motor symptoms or provide official diagnosis documents during participation. Therefore, the “suspected PD” category may include individuals in the prodromal phase of PD, individuals with other motor or neurological conditions, or even individuals without any actual motor conditions.

Participants used a physical mouse on their desktop or laptop to trace a straight line, sine wave, and spiral wave on the website. We visualized their progress and alignment with the lines through highlighted portions and start or end markings. We developed the website using HTML and Bootstrap for the interface and visuals, and JavaScript to track cursor position every 500 ms. The data included mouse positions (x and y axes) and whether the mouse was inside the line (true or false). The web application also captured screen dimensions and operating system details for contextual information. Upon completing the test, all data were securely transmitted and stored in a Google Firebase collection.

### Feature Engineering and Mouse-Trace Image Generation

Based on the raw data collected, we engineered several features to quantify tracing performance across the 3 tracing tasks (straight line, sine wave, and spiral). These features include the following:

- *Mean deviation from centerline (straight line only)*: the average absolute vertical deviation of the traced points from the expected centerline, normalized by the screen height. The centerline is determined based on the screen height and adjusted to align with the web interface.
- *Time taken (ms)*: the total tracing time is approximated by multiplying the number of recorded points by the 500-ms sampling interval.
- *Time taken with respect to window width (ms/pixel)*: the total time, normalized by the screen width, is calculated as the total time divided by the window width. This metric accounts for variations in screen size among participants.
- *Percentage of points traced inside the target*: the proportion (from 0 to 1) of recorded points that fall inside the expected trace area.
- *Raw count of points traced inside the target*: simply the number of points recorded inside the expected trace area.

Using the features shown in Table 1, we quantified the consistency and accuracy of a participant’s trace on each test pattern.

**Figure 3.**
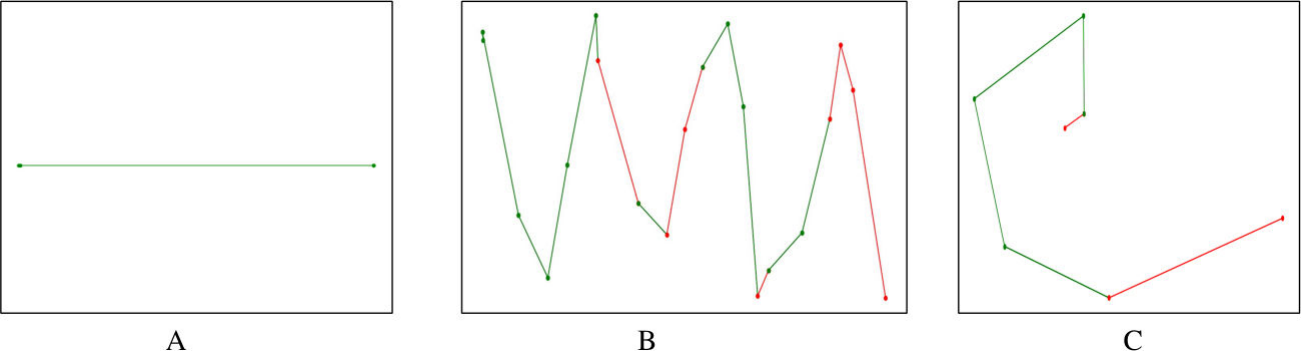
Samples of mouse-trace images produced: (A) Straight line; (B) Sine wave; (C) Spiral wave.

To generate mouse-trace images, we created canvases corresponding to each participant’s screen dimensions. The recorded x and y coordinates were then used to render the trace, where segments starting with points inside the target region are colored green and those that fall outside are colored red. Figure 3 illustrates the samples of these plots.

**Table 1.** Engineered features extracted from mouse-trace data.

Feature name	Description	Applicable trace
Mean deviation from centerline (fraction of screen height)	Average absolute vertical deviation from the expected centerline, normalized by the screen height	Straight line
Time taken (ms)	Total time estimated by multiplying the number of recorded points by 500 ms	All
Time taken with respect to window width (ms/pixel)	Total time divided by the window width. This normalizes the tracing duration to account for differences in screen dimensions	All
Percentage of points traced inside (as a fraction, 0-1)	Percentage of the number of points inside the expected trace region to the total number of points	All
Number of points traced inside	Count of points that are within the expected trace region, irrespective of timing	All

### Model Selection and Model Development

**Figure 4.**
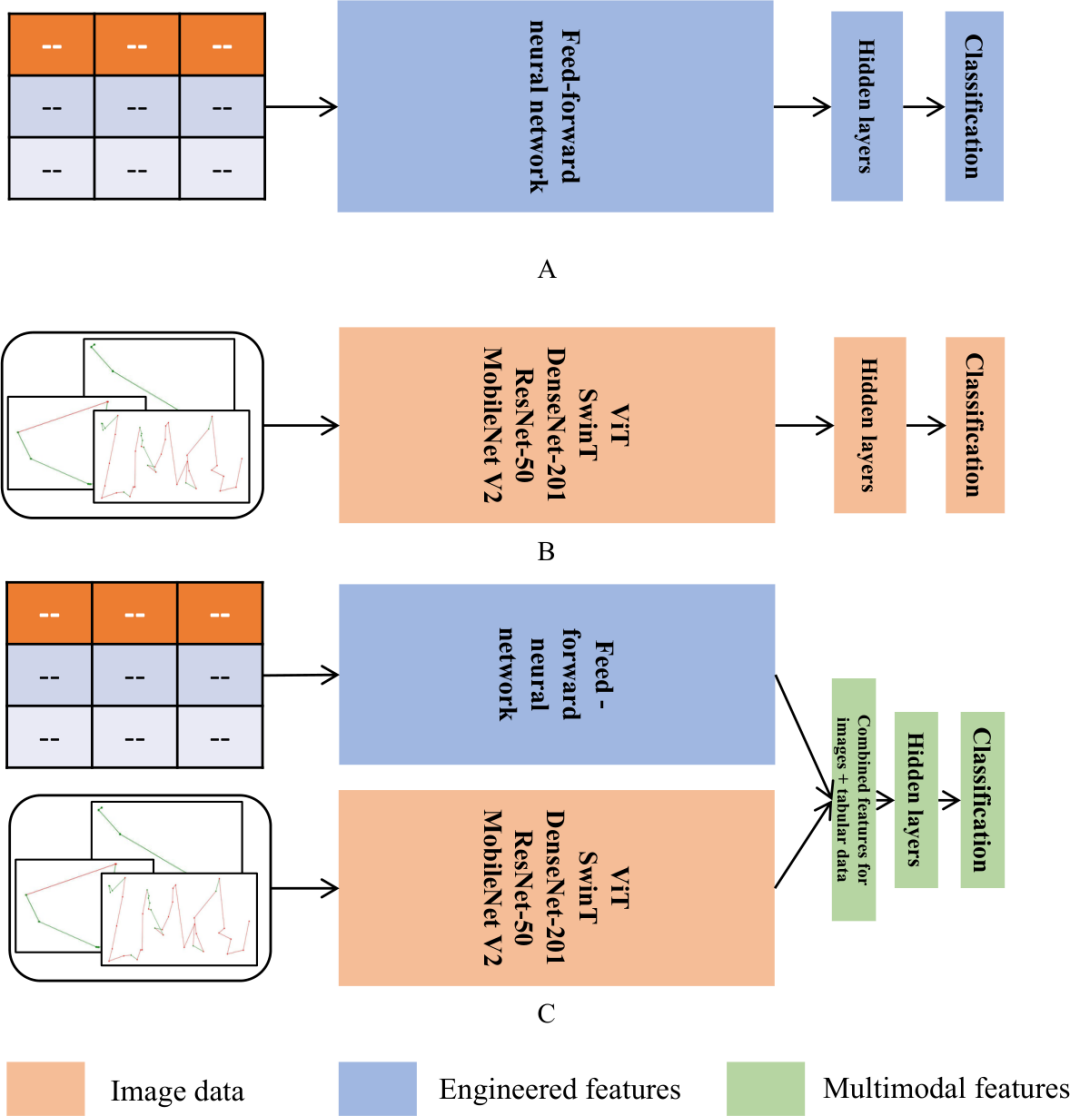
Model architectures. (A) Tabular data models; (B) Image data models; (C) Multimodal models. DenseNet-201: Densely Connected Convolutional Network-201; MobileNet V2: Mobile Network v2; ResNet-50: Residual Network-50; SwinT: Shifted Window Transformer; ViT: Vision Transformer.

We developed three categories of deep-learning models, as shown in Figure 4, each utilizing different input modalities, to better understand the individual and combined contributions of image and tabular mouse-trace data for PD classification. The first category of models focused on engineered tabular features, processed through a feed-forward neural network. We used this category to determine whether using purely engineered features can reliably classify patients diagnosed with PD. A feed-forward neural network was selected for consistency with the tabular data branch used later in the multimodal models. The second category of models comprised image-based models, where input images were passed through pretrained convolutional neural network (CNN)–based architectures: Densely Connected Convolutional Network-201 (DenseNet-201), Residual Network-50 (ResNet-50), and Mobile Network v2 (MobileNet V2), and two transformer-based architectures: Vision Transformer (ViT) and Shifted Window Transformer (SwinT). CNN-based architectures (ie, DenseNet-201, ResNet-50, and MobileNet V2) excel at capturing edges and textures, which can help detect tremor-related discrepancies in the image trace. On the other hand, transformer-based architectures such as ViT use global self-attention, which can help capture the overall geometry of the traces. Additionally, SwinT uses hierarchical shifting-window attention, which can help capture both fine-grained patterns and the overall trace shape. Specifically, we used ViT-Base-Patch16-224 and swin_base_patch4_window7_224. Each of these models was selected based on their prior performance on different image-based tasks, as both CNN and transformer-based architectures have demonstrated tolerance to variations in scale, resolution, and local noise—properties that are expected in remotely collected mouse-trace images generated from diverse hardware and screen configurations. Transfer learning was applied by unfreezing the final 45 layers of each network and by replacing the classification heads for training. The third category of models consisted of multimodal models, which integrated both data types. Tabular features were passed through a feed-forward neural network, while image inputs were processed via the same pretrained backbones used in the image-based models. The resulting feature representations were concatenated and passed through additional hidden layers for classification. All models were trained as binary classifiers for up to 50 epochs, with early stopping applied using a patience of 5. By structuring the models consistently across these categories, we aimed to isolate and analyze the impact of each data modality and understand how individual components contribute to the performance of the multimodal architectures.

### Data Splitting and Hyperparameter Tuning

We evaluated model performance across 3 experimental settings, with 1 primary experiment and 2 secondary analyses, designed to reflect different levels of diagnostic certainty and real-world deployment scenarios. The primary experiment focuses on assessing the feasibility of training models on patients who self-reported suspected PD and predicting diagnosed PD.

In the primary experiment, models were trained on participants with suspected PD and 60% of non-PD data and evaluated on all PD cases and the remaining 40% of non-PD samples, split equally into a test set and a validation set. This experiment directly addresses the central research objective by assessing whether models trained on low-specificity, community-recruited labels can generalize to predict diagnosed PD. To mitigate variability caused by nondeterministic behavior observed in some of our image-based and multimodal models, final performance was estimated by averaging results across 20 independently trained models, with average performance reported using 500 bootstrap resamples in each run. Model hyperparameters were tuned using Optuna [46] over 30 trials.

In a secondary analysis, models were trained on all participants with self-reported PD and 60% of the samples without PD and evaluated on participants with suspected PD combined with the remaining 40% of non-PD data. This analysis was designed to assess whether models trained on patients diagnosed with PD can generalize to individuals with uncertain or emerging motor symptoms. Hyperparameters were tuned using Optuna over 30 trials, and final performance was estimated by averaging results across 20 independently trained models, each evaluated using 500 bootstrap resamples. This analysis is not part of the primary study objective and is included only to assess whether the learned signal is bidirectional.

In the cross-validation analysis, we included only participants labeled as either self-reported PD or non-PD, explicitly excluding those marked as suspected PD. Models were trained and evaluated using 5-fold cross-validation. Within each fold, hyperparameters were tuned using Optuna across 10 trials, and performance was estimated using 500 bootstrapped resamples per fold. We repeated each fold 10 times with independently initialized models, reporting average performance across runs. This experiment established a baseline for distinguishing controls with PD from controls without PD using stronger supervisory labels.

Bootstrapping across the different experiments was used to estimate variability and model stability under resampling. However, these approaches do not increase the amount of independent data and therefore do not fully capture uncertainty arising from the limited dataset size.

### Evaluation Metrics

To evaluate model performance, we used 4 commonly used classification metrics: sensitivity, specificity, accuracy, and *F*_1_-score. These metrics were calculated using the confusion matrix derived from model predictions, consisting of the following:

- True positives: cases correctly predicted as positive
- True negatives: cases correctly predicted as negative
- False positives: negative cases incorrectly predicted as positive
- False negatives: positive cases incorrectly predicted as negative

From these values, we calculated the following metrics:

- Sensitivity, the proportion of actual positive cases correctly identified, is defined as $\text{sensitivity}=\frac{TP}{TP+FN}$.
- Specificity, the proportion of actual negative cases correctly classified, is defined as $\text{specificity}=\frac{TN}{TN+FP}$.
- Accuracy, the overall proportion of correctly classified cases across both classes, is calculated as  $\text{accuracy}=\frac{TP+TN}{TP+TN+FP+FN}$.
- The *F*_1_-score, the harmonic mean of precision and recall, is particularly useful for imbalanced class distributions. The *F*_1_-score is computed as $F_{1}\text{-score}=2\times \frac{\text{Precision}\times \text{Recall}}{\text{Precision}+\text{Recall}}$, where $\text{precision}=\frac{TP}{TP+FP}$ and $\text{recall}=\frac{TP}{TP+FN}$.

Each of these metrics was estimated using 500 bootstrapped resamples to compute both the mean and SD, providing a robust measure of model stability and generalizability. We used the mean *F*_1_-score as the base metric to select the best performing models from our analysis. All metric values that we presented included the mean and SD of the metrics.

### Saliency Map and Feature Importance

To identify important features for the best performing model architectures, we created GradSHAP (Gradient Shapley Additive Explanations) saliency plots, as GradSHAP is optimized for identifying complex feature importances in deep-learning models [47]. GradSHAP was applied to both the image and engineered tabular inputs to quantify their respective contributions to the model’s predictions. For the image modality, attributions were computed separately for the sine, straight, and spiral images by comparing the model’s output on actual inputs and shuffled versions of the same batch, used as baselines. Attribution values were summed across spatial dimensions to yield a single importance score for each image input. Importantly, the sign of each attribution was preserved to indicate the direction of influence: positive values reflect contributions toward a PD or suspected PD prediction, while negative values indicate support for a non-PD classification.

### Ethical Considerations

Our study was approved by the University of Hawaii Institutional Review Board (protocol 2022‐00857). We collected informed consent forms from all participants at the start of data collection on the website. All data used in this analysis were anonymized by default, as the assessment data did not include any personal information.

## Results

### Dataset

**Figure 5.**
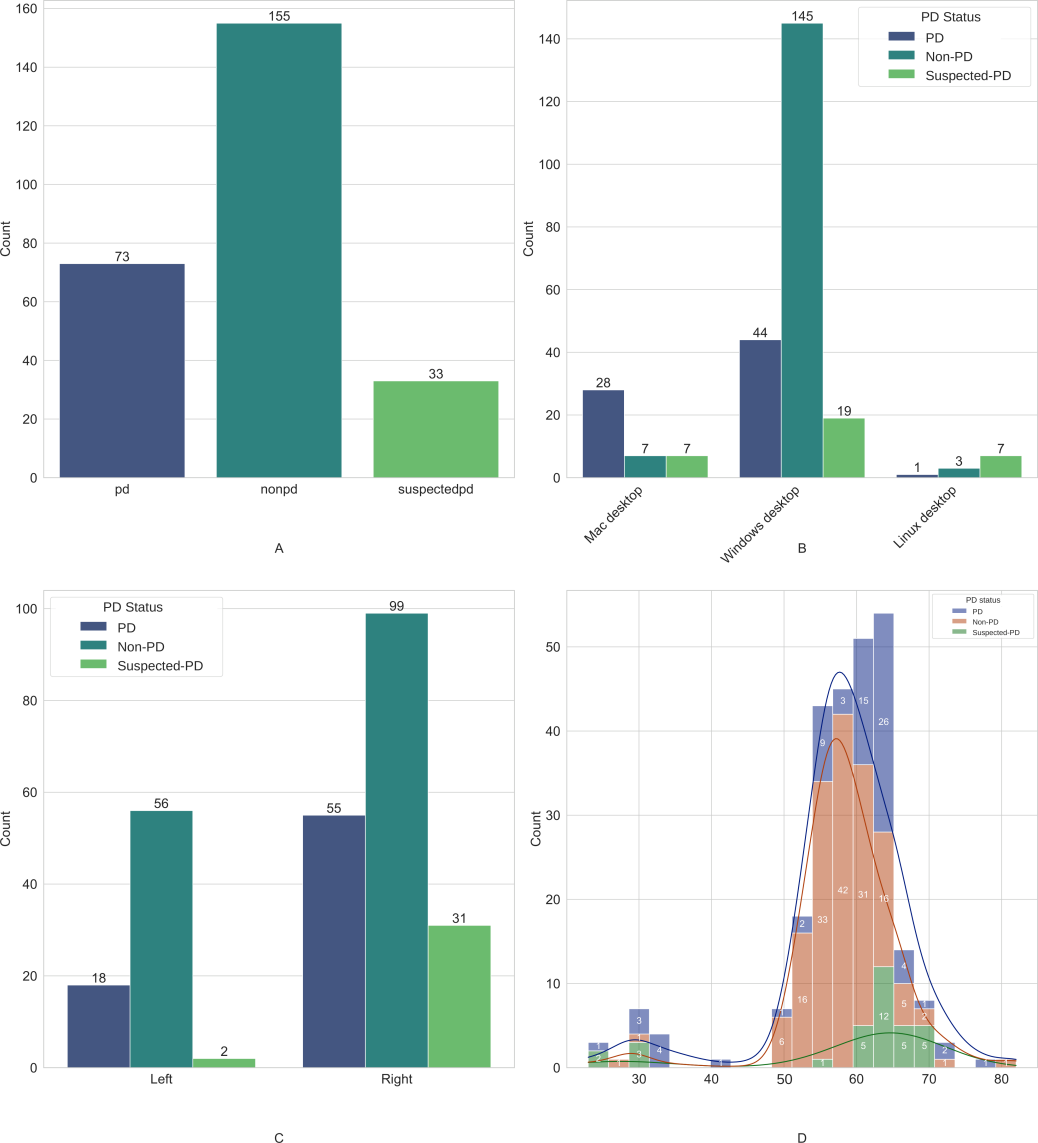
Participant distribution among the different classes: (A) Parkinson disease (PD), non-PD, and suspected PD, stratified by (B) device type, (C) dominant hand, and (D) age.

We had 261 participants with completed attempts for the mouse-trace section of the website. Among the 261 participants, 73 self-reported as having PD, 155 as non-PD, and the remaining 33 as suspected PD. Attempts with data available for all 3 mouse-trace tasks, irrespective of the age group, were included. The characteristics of the collected data are shown in Figure 5 and Table 2.

**Table 2.** Participant distribution among the different classes (Parkinson disease [PD], non-PD, and suspected PD) for age, dominant hand, and device type.

Characteristics	Participants	PD	Non-PD	Suspected PD
Age (y)				
23‐49	16	9	2	5
50‐59	113	15	97	1
60‐69	126	46	53	27
70+	6	3	3	0
Dominant hand				
Right	185	55	99	31
Left	76	18	56	2
Device type				
Windows	208	44	145	19
Mac	42	28	7	7
Linux	11	1	3	7
Total	261	73	155	33

### Performance Metrics

#### Primary Experiment: Training on Suspected PD vs Non-PD and Testing on PD vs Non-PD

**Figure 6.**
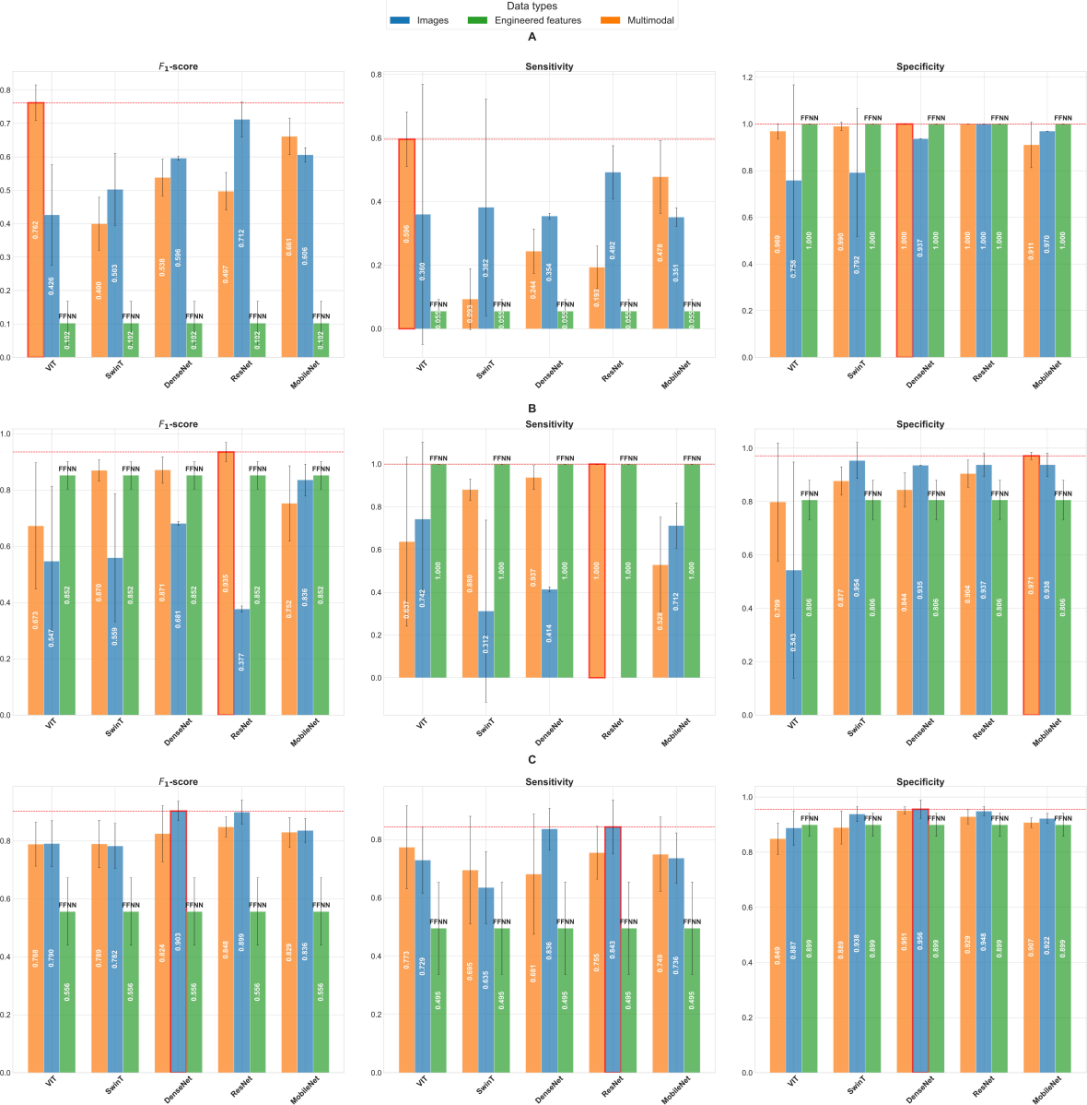
Key performance metrics (*F*_1_-score, sensitivity, and specificity) for all models. (A) Trained on suspected Parkinson disease (PD) versus non-PD and tested on PD versus non-PD data evaluated with 500 bootstraps resampling. (B) Trained on PD versus non-PD and tested on suspected PD versus non-PD data. (C) Trained and tested on PD versus non-PD data (on 5-fold cross-validation). The error bars represent SD values. DenseNet: densely connected convolutional network; FFNN: feed-forward neural network; MobileNet: Mobile Network; ResNet: Residual Network; SwinT: Shifted Window Transformer; ViT: Vision Transformer.

In our primary experiment, where models trained on suspected PD and non-PD data were tested on patients diagnosed with PD and without PD, the multimodal ViT model performed best, achieving a mean *F*_1_-score of 0.7619 (SD 0.0535) with balanced performance (accuracy: mean 0.7663, SD 0.0500; sensitivity: mean 0.5961, SD 0.0859; specificity: mean 0.9694, SD 0.0319). Overall, the performance improved as we moved from tabular to image data: the feed-forward neural network (*F*_1_-score: mean 0.1022, SD 0.0654) achieved only 0.0551 (SD 0.0369) in sensitivity but 1.0000 (SD 0.0000) in specificity, indicating severe overfitting due to class imbalance. Looking at image-based models, ResNet-50 led the group (*F*_1_: mean 0.7119, SD 0.0529; accuracy: mean 0.7236, SD 0.0455; sensitivity: mean 0.4921, SD 0.0836; specificity: mean 1.0000, SD 0.0000), while DenseNet-201 and MobileNet V2 performed more poorly (DenseNet-201 *F*_1_: mean 0.5958, SD 0.0062; MobileNet V2 *F*_1_: mean 0.6059, SD 0.0216). Adding engineered features to the image backbones produced mixed results for the *F*_1_-score: ViT’s *F*_1_-score jumped from 0.4263 (SD 0.1504) to 0.7619 (SD 0.0535) and MobileNet V2 went from 0.6059 (SD 0.0216) to 0.6611 (SD 0.0549), but the *F*_1_-score for DenseNet201, SwinT, and ResNet-50 actually dropped (DenseNet-201: from 0.5958, SD 0.0062 to 0.5383, SD 0.0553; SwinT: from 0.5026, SD 0.1076 to 0.3998, SD 0.0792; and ResNet-50: from 0.7119, SD 0.0529 to 0.4970, SD 0.0563). These results suggest that models trained on suspected PD samples can generalize to actual self-reported cases better than chance and that multimodal fusion provides meaningful gains for some backbones but not all. Complete performance metrics for this split are shown in Table 3 and Figure 6A.

**Table 3.** The performance metrics for all models trained on suspected Parkinson disease (PD) and non-PD data, tested on PD and non-PD data with 500 bootstrap resampling.

Data type and model	Accuracy, mean (SD)	Sensitivity, mean (SD)	Specificity, mean (SD)	*F*_1_, mean (SD)
Multimodal				
ViT^a^	0.7663 (0.0500)	0.5961 (0.0859)	0.9694 (0.0319)	0.7619 (0.0535)
SwinT^b^	0.5023 (0.0477)	0.0934 (0.0960)	0.9902 (0.0182)	0.3998 (0.0792)
DenseNet-201^c^	0.5886 (0.0383)	0.2438 (0.0703)	1.0000 (0.0000)	0.5383 (0.0553)
ResNet-50^d^	0.5610 (0.0365)	0.1931 (0.0671)	1.0000 (0.0000)	0.4970 (0.0563)
MobileNet V2^e^	0.6752 (0.0444)	0.4778 (0.1149)	0.9108 (0.0985)	0.6611 (0.0549)
Images				
ViT	0.5415 (0.1018)	0.3599 (0.4091)	0.7582 (0.4094)	0.4263 (0.1504)
SwinT	0.5686 (0.0702)	0.3816 (0.3403)	0.7919 (0.2737)	0.5026 (0.1076)
DenseNet-201	0.6199 (0.0049)	0.3544 (0.0089)	0.9369 (0.0000)	0.5958 (0.0062)
ResNet-50	0.7236 (0.0455)	0.4921 (0.0836)	1.0000 (0.0000)	0.7119 (0.0529)
MobileNet V2	0.6330 (0.0159)	0.3510 (0.0292)	0.9695 (0.0000)	0.6059 (0.0216)
Engineered features				
FFNN^f^	0.4859 (0.0201)	0.0551 (0.0369)	1.0000 (0.0000)	0.1022 (0.0654)

a ViT: Vision Transformer.

b SwinT: Shifted Window Transformer.

c DenseNet-201: Densely Connected Convolutional Network-201.

d ResNet-50: Residual Network-50.

e MobileNet V2: Mobile Network v2.

f FFNN: feed-forward neural network.

#### Secondary Analysis: Training on PD vs Non-PD, Testing on Suspected PD vs Non-PD

In one of our secondary analyses, models were trained on PD and non-PD data and tested on suspected PD and non-PD data. The multimodal ResNet-50 model achieved the highest *F*_1_-score (mean 0.9353, SD 0.0334), with balanced sensitivity (mean 1.0000, SD 0.0000) and specificity (mean 0.9045, SD 0.0506). The feed-forward neural network using only engineered features scored a mean *F*_1_ of 0.8523 (SD 0.0488; accuracy: mean 0.8746, SD 0.0474; sensitivity: mean 1.0000, SD 0.0000; specificity: mean 0.8058, SD 0.0734), showing that tabular data alone still carry useful signals but may not match multimodal models in this case. Among the image-based models, MobileNet V2 was the best, with a mean *F*_1_-score of 0.8359 (SD 0.0560; accuracy: mean 0.8575, SD 0.0470; sensitivity: mean 0.7118, SD 0.1058; specificity: mean 0.9375, SD 0.0443). DenseNet-201 followed with mean *F*_1_ of 0.6809 (SD 0.0073; accuracy: mean 0.7507, SD 0.0043; sensitivity: mean 0.4137, SD 0.0122; specificity: mean 0.9355, SD 0.0000), while SwinT and ViT trailed (SwinT *F*_1_: mean 0.5588, SD 0.2272; ViT *F*_1_: mean 0.5467, SD 0.2659). Notably, ResNet-50 trained on images failed to detect any positives (sensitivity: mean 0.0000, SD 0.0000) and scored mean *F*_1_ of 0.3769 (SD 0.0108). Adding engineered features with image inputs produced positive effects for the *F*_1_-score: ResNet-50 jumped from mean 0.3769 (SD 0.0108) to mean 0.9353 (SD 0.0334); DenseNet-201 jumped from mean 0.6809 (SD 0.0073) to mean 0.8712 (SD 0.0472); SwinT jumped from mean 0.5588 (SD 0.2272) to mean 0.8697 (SD 0.0380); and ViT went from mean 0.5467 (SD 0.2659) to mean 0.6726 (SD 0.2242). By contrast, the *F*_1_-score of MobileNet V2 dipped from mean 0.8359 (SD 0.0560) to mean 0.7523 (SD 0.1336). These results indicate that while detecting early or ambiguous motor patterns in suspected PD, multimodal fusion can improve robustness and performance balance. Complete performance metrics for this split are shown in Table 4, and comparisons of *F*_1_-score, sensitivity, and specificity are visualized in Figure 6B.

**Table 4.** The performance metrics for models trained on Parkinson disease (PD) and non-PD data, tested on suspected PD and non-PD data with 500 bootstrap resampling.

Data type and model	Accuracy, mean (SD)	Sensitivity, mean (SD)	Specificity, mean (SD)	*F*_1_, mean (SD)
Multimodal				
ViT^a^	0.7414 (0.1585)	0.6370 (0.3958)	0.7986 (0.2211)	0.6726 (0.2242)
SwinT^b^	0.8782 (0.0369)	0.8801 (0.0501)	0.8771 (0.0528)	0.8697 (0.0380)
DenseNet-201^c^	0.8768 (0.0465)	0.9371 (0.0561)	0.8438 (0.0645)	0.8712 (0.0472)
ResNet-50^d^	0.9383 (0.0327)	1.0000 (0.0000)	0.9045 (0.0506)	0.9353 (0.0334)
MobileNet V2^e^	0.8145 (0.0747)	0.5285 (0.2227)	0.9713 (0.0138)	0.7523 (0.1336)
Images				
ViT	0.6135 (0.2308)	0.7422 (0.3615)	0.5430 (0.4047)	0.5467 (0.2659)
SwinT	0.7263 (0.1106)	0.3119 (0.4256)	0.9535 (0.0675)	0.5588 (0.2272)
DenseNet-201	0.7507 (0.0043)	0.4137 (0.0122)	0.9355 (0.0000)	0.6809 (0.0073)
ResNet-50	0.6054 (0.0274)	0.0000 (0.0000)	0.9374 (0.0424)	0.3769 (0.0108)
MobileNet V2	0.8575 (0.0470)	0.7118 (0.1058)	0.9375 (0.0443)	0.8359 (0.0560)
Engineered features				
FFNN^f^	0.8746 (0.0474)	1.0000 (0.0000)	0.8058 (0.0734)	0.8523 (0.0488)

a ViT: Vision Transformer.

b SwinT: Shifted Window Transformer.

c DenseNet-201: Densely Connected Convolutional Network-201.

d ResNet-50: Residual Network-50.

e MobileNet V2: Mobile Network v2.

f FFNN: feed-forward neural network.

#### Cross-Validation Analysis: 5-Fold Cross-Validation Predicting PD vs Non-PD

We also conducted a cross-validation analysis using PD and non-PD data for both training and testing via 5-fold cross-validation. The image-based DenseNet-201 model achieved the highest *F*_1_-score (mean 0.9027, SD 0.0332), alongside an accuracy of 0.9179 (SD 0.0288), a sensitivity of 0.8360 (SD 0.0713), and a specificity of 0.9557 (SD 0.0327). Overall, the pretrained CNNs (ResNet-50 *F*_1_: mean 0.8986, SD 0.0413; MobileNet V2 *F*_1_: mean 0.8355, SD 0.0410; and DenseNet-201 *F*_1_: mean 0.9027, SD 0.0332) outperformed both transformer-based image models (ViT *F*_1_: mean 0.7900, SD 0.0789; SwinT *F*_1_: mean 0.7819, SD 0.0774) and the feed-forward neural network using only engineered features (*F*_1_: mean 0.5558, SD 0.1161), which showed high specificity (mean 0.8993, SD 0.0419) but low sensitivity (mean 0.4955, SD 0.1583). When tabular features were added to the image data, multimodal SwinT led to a negligibly small *F*_1_ gain (around +0.0071). By contrast, all other multimodal models experienced *F*_1_ drops of approximately 0.001 to 0.0785. These results suggest that while image-based CNNs capture the majority of diagnostic signals from mouse-trace images, the marginal benefit of integrating engineered features is almost nonexistent for most of the architectures. Complete performance metrics for this split are provided in Table 5 and Figure 6C.

**Table 5.** The performance metrics for models trained and tested on Parkinson disease (PD) and non-PD data, evaluated using 5-fold cross-validation with 500 bootstrap resampling.

Data type and model	Accuracy, mean (SD)	Sensitivity, mean (SD)	Specificity, mean (SD)	*F*_1_, mean (SD)
Multimodal				
ViT^a^	0.8255 (0.0529)	0.7730 (0.1422)	0.8488 (0.0566)	0.7883 (0.0757)
SwinT^b^	0.8263 (0.0608)	0.6949 (0.1847)	0.8891 (0.0595)	0.7890 (0.0822)
DenseNet-201^c^	0.8654 (0.0638)	0.6809 (0.2060)	0.9507 (0.0134)	0.8242 (0.0968)
ResNet-50^d^	0.8727 (0.0260)	0.7547 (0.0905)	0.9286 (0.0266)	0.8476 (0.0341)
MobileNet V2^e^	0.8566 (0.0367)	0.7492 (0.1275)	0.9071 (0.0175)	0.8294 (0.0513)
Images				
ViT	0.8366 (0.0545)	0.7290 (0.1128)	0.8875 (0.0619)	0.7900 (0.0789)
SwinT	0.8404 (0.0421)	0.6352 (0.1230)	0.9379 (0.0270)	0.7819 (0.0774)
DenseNet-201	0.9179 (0.0288)	0.8360 (0.0713)	0.9557 (0.0327)	0.9027 (0.0332)
ResNet-50	0.9141 (0.0329)	0.8430 (0.0917)	0.9484 (0.0168)	0.8986 (0.0413)
MobileNet V2	0.8629 (0.0319)	0.7359 (0.0865)	0.9220 (0.0183)	0.8355 (0.0410)
Engineered features				
FFNN^f^	0.7689 (0.0432)	0.4955 (0.1583)	0.8993 (0.0419)	0.5558 (0.1161)

a ViT: Vision Transformer.

b SwinT: Shifted Window Transformer.

c DenseNet-201: Densely Connected Convolutional Network-201.

d ResNet-50: Residual Network-50.

e MobileNet V2: Mobile Network v2.

f FFNN: feed-forward neural network.

### Feature Importance Analysis of Multimodal Models

We selected the best-performing models from each experimental setting based on the highest mean *F*_1_-scores achieved across runs. In the primary experiment, the multimodal ViT achieved the best performance, and in the secondary analysis, the multimodal ResNet-50 performed best. In the cross-validation analysis, the image-based DenseNet-201 model achieved the highest performance.

We conducted GradSHAP-based feature importance analysis on the 2 multimodal models from the primary experiment and the secondary analysis to better understand the relative contributions of image and engineered features. We did not perform the feature importance analysis on the image-based DenseNet-201 model, as such an analysis would provide limited additional insight: with no tabular features present, the dominance of image inputs is expected and not subject to comparison. Our goal was to explore how much influence tabular features or image features have in a multimodal setting and whether models rely solely on image inputs or leverage both data types.

**Figure 7.**
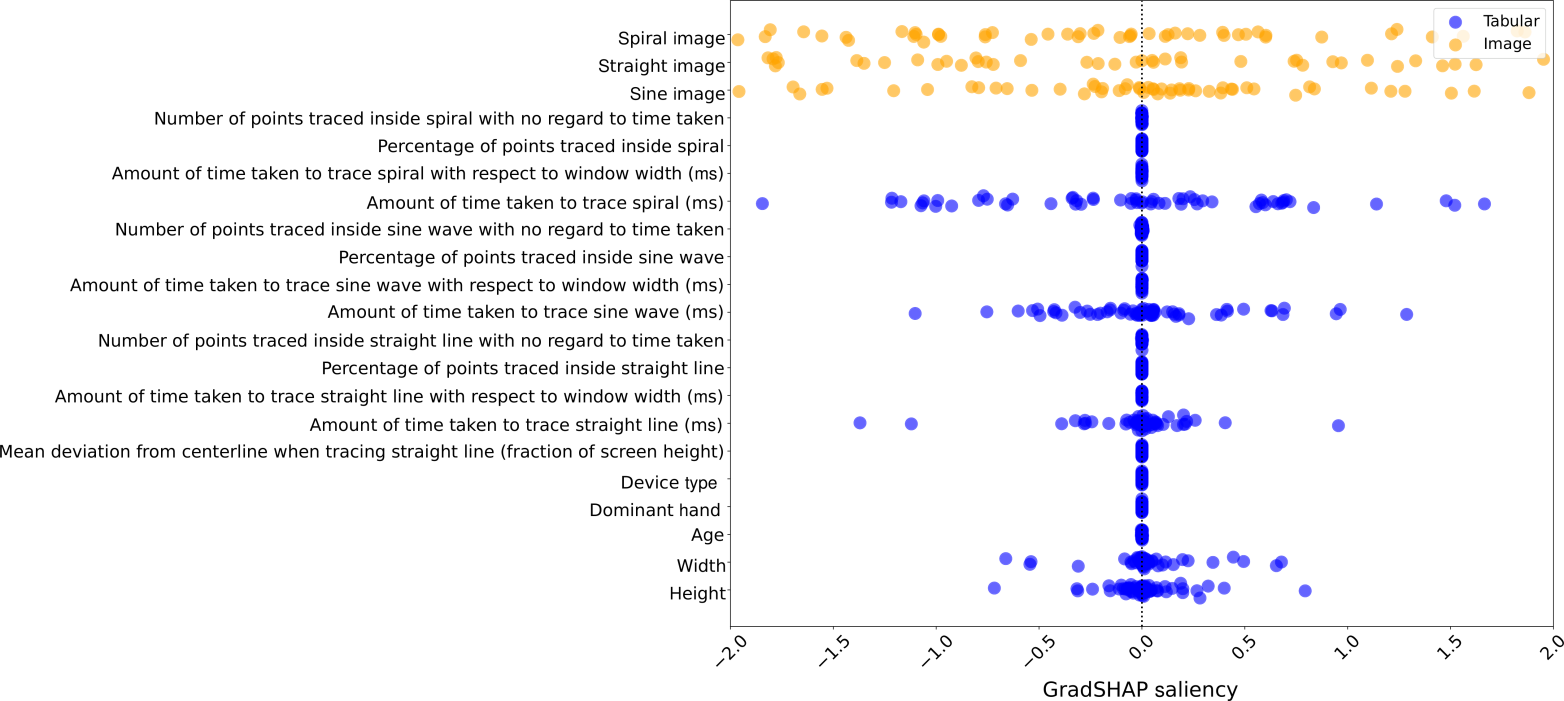
Interpretability analysis of the multimodal Vision Transformer (ViT) model for the patients with self-reported Parkinson disease (PD) and without PD (trained on suspected PD and non-PD). (The x-axis range is set to [−2, 2] in the figure for better comparison, and the outlier GradSHAP [Gradient Shapley Additive Explanations] values are removed from the figure.)

Figure 7 depicts the feature importance analysis for the multimodal ViT model trained on suspected PD versus non-PD and tested on PD versus non-PD. We found that the model relied heavily on the images for prediction. However, in this case, the engineered features, especially the time required to trace the patterns, exhibited high SHAP (Shapley Additive Explanations) values. The height and width of the screen used by the participant also exhibited relatively high SHAP values.

**Figure 8.**
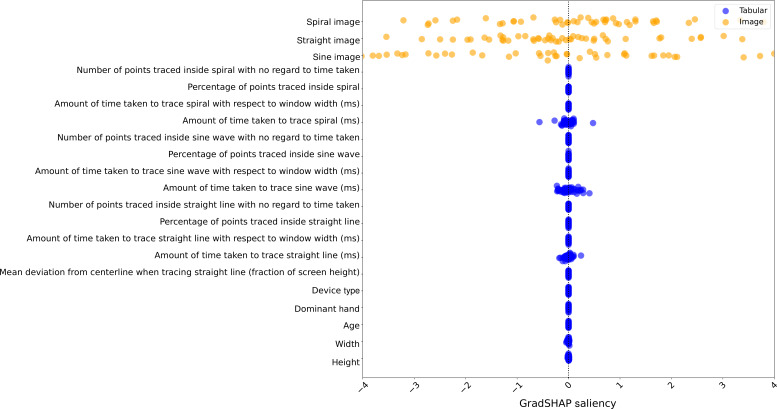
Interpretability analysis of the multimodal ResNet-50 (Residual Network-50) model for the patients with self-reported suspected Parkinson disease (PD) and without PD (trained on PD and non-PD). (The x-axis range is set to [−4, 4] in the figure for better comparison, and the outlier GradSHAP [Gradient Shapley Additive Explanations] values are removed from the figure.)

Figure 8 depicts the feature importance analysis for the multimodal ResNet-50 model trained on PD versus non-PD and tested on suspected PD versus non-PD. We found that the model relied predominantly on image features for prediction, with the spiral, sine, and straight line images showing the highest SHAP values. Among the engineered features, only the time taken to trace the spiral and sine waves, as well as the straight line, exhibited significant SHAP values, while the rest of the tabular features had a negligible impact.

## Discussion

### Principal Results

We sought to study the feasibility of training models using weak labels of “suspected PD” to predict confirmed PD in a community-recruited sample. We evaluated model performance using 1 primary experiment and 2 secondary analyses, each reflecting different assumptions about diagnostic certainty and real-world deployment scenarios. The primary experiment involved training models on participants with suspected PD and without PD and evaluating them on patients with self-reported PD and without PD, reflecting a real-world use case in which models trained on low-specificity, community-recruited labels are applied to identify diagnosed PD. We found that training models on patients with suspected PD yielded moderate yet meaningful performance in predicting patients with self-reported PD. These preliminary findings support the feasibility of leveraging community-recruited, low-specificity labels to learn patterns that generalize to predicting diagnosed PD. This suggests that, even in the presence of diagnostic uncertainty in labels, suspected PD data can possibly retain signals relevant to motor symptoms that may still be useful for developing scalable screening tools in global health contexts. However, we emphasize that this claim needs to be supported by future work involving participants with clinically confirmed diagnoses.

We also found that images derived from spiral, sine, and straight line tracing were the dominant contributors to model predictions for the multimodal models. Tabular features such as tracing time showed modest attribution in the ResNet-50 (secondary analysis), while showing much higher influence in ViT (primary analysis with suspected PD). Device-related features (screen height and width) played a minimal to nonexistent role. In the ViT model trained on patients with suspected PD, the increased influence of engineered features suggests that engineered data can complement visual patterns when used to train models to identify real patients with PD.

### Comparison to Previous Works

To the best of our knowledge, we conducted the first pilot feasibility study to assess whether lower-specificity weak labels of “suspected PD” can enable artificial intelligence models to learn useful motor signatures related to PD. From a computational methods perspective, our study also expands upon previous research by introducing an approach relying exclusively on mouse-trace data and images produced from a brief online test for PD classification through the use of deep-learning models. Unlike prior methods that have explored hand and finger movements, keyboard typing patterns, keystroke dynamics, speech analysis, handwriting, drawing tests, and sensor data from accelerometers, gyroscopes, and smartphone interactions, we specifically focus on the remote collection of mouse-tracing data, and our experiments demonstrate the potential of mouse-trace data collected from a heterogeneous array of devices to provide predictive power in classifying PD.

Previous studies by Gil-Martin et al [48] and Pereira et al [49] focused on hand movement dynamics from spiral, meander, and other drawing shapes for PD analysis. However, their data collection was not conducted remotely, and they did not consider handedness, unlike our study. Their best models achieved accuracies of 97.7% and 83.77%, respectively. Goel et al [50] used pen-and-paper methods to collect spiral pattern data but also lacked remote testing and consideration of handedness, achieving an accuracy of 87.36%. Memedi et al [51] used a remote data collection method involving a touchscreen tablet and web interface, but their study spanned 3 years and involved only 65 participants, resulting in an accuracy of around 84%. Wang et al [52] achieved 96.2% accuracy using the data collected during the drawing of Π and ΠΛ shapes. However, their data collection method involved an iPad and stylus and was not conducted remotely. The classification model suggested by Gajos et al [53] detected ataxia or parkinsonism with high sensitivity (0.91) and specificity (0.90) but included only 46 patients with PD in their dataset with 22,946 controls.

These studies served as important and inspirational foundational literature that we aimed to build upon. The results from most prior works were obtained under controlled conditions with specialized hardware; however, our remote approach trades some performance for scalability.

A previous pilot study of ours [54], which explored the feasibility of an earlier version of our web application on a much smaller participant sample, achieved an accuracy of 74.29% and an *F*_1_-score of 73.11% using classical machine-learning models. This work used both keyboard and mouse-trace data as engineered features and did not include patients with suspected PD in the data collection process. Our current models show marked improvements in performance compared to this prior pilot study, possibly due to either our novel machine-learning approach presented here or the significantly expanded dataset size.

### Clinical Implications and Further Validation

Our findings support 2 key observations. First, despite label noise, models trained using patients with “suspected PD” may retain sufficient motor signals to enable the meaningful prediction of diagnosed PD, supporting the feasibility of using community-recruited, low-specificity data for model development. Second, motor patterns observed in individuals with suspected PD share detectable similarities with those diagnosed with PD, allowing the models to generalize even when diagnostic certainty is low. However, several key validation steps are required to fully validate this approach, including evaluation using clinically confirmed prodromal PD cohorts and the inclusion of other related motor disorders for a more rigorous assessment of model specificity (ie, whether the models are actually identifying PD or reporting noise caused by motor dysfunctions). Ultimately, our findings should be interpreted as preliminary feasibility evidence to support further, larger-scale investigation.

### Limitations and Future Work

As a pilot feasibility study, this work has several limitations that should be considered in future research. First, the sample size of 261, split into 3 diagnostic categories, may not be sufficient to generalize the findings across a diverse population and heterogeneous disease stages. This limitation is particularly relevant for the suspected PD group, which contained only 33 participants. Although we tried to mitigate this through repeated training runs and evaluation across multiple splits, small sample sizes can still affect model generalizability and may have been the reason behind the occasional appearance of extreme values for sensitivity and specificity. Moreover, this small sample size for the suspected PD group limits the statistical power and generalizability of our findings. In the future, we plan to prioritize expanding recruitment and validating these findings in larger and more diverse cohorts. Moreover, while we performed hyperparameter tuning for each model, further optimization, especially regarding model architecture and data fusion strategies, could improve performance and generalization. Some models, particularly those involving image-based and multimodal inputs, exhibited nondeterministic behavior. These models produced variable results across repeated training runs on identical data splits, likely due to the stochastic nature of deep learning, their sensitivity to complex, high-dimensional input spaces, and resampling on the small sample sizes. This reinforces the importance of repeated experimentation and performance averaging in evaluating model robustness. We used a fixed sampling rate of 500 ms to store mouse positions on screen, resulting in some data points with only one time step represented. While we retained those data in this study, future work should explore more granular sampling rates (eg, 50/100 ms) and stricter exclusion criteria.

Notably, a key limitation of this study is that the “suspected PD” category was based solely on self-report and did not require clinical verification. Consequently, this group is heterogeneous and may include individuals with health anxiety, age-related motor conditions, or non-PD neurodegenerative disorders. Future work should incorporate longitudinal follow-up or clinician-verified diagnoses to better characterize which patients with suspected PD progress to PD and to refine early-stage prediction models.

Additionally, our focus on mouse-tracing data collected through a website does not fully capture all aspects of PD symptoms. Importantly, the study did not account for medication usage, specifically distinguishing the “on” phase versus the “off” phase, which can significantly influence the presence and severity of symptoms such as tremors [55-57]. Stress, which is known to exacerbate tremors, was also not accounted for, potentially affecting the results [58]. All data were collected remotely using participants’ own devices, resulting in variability in devices, operating systems, and screen configurations. Participant characteristics, such as age and handedness, were not balanced across groups. These factors may introduce confounding effects, as differences in hardware, cursor behavior, or user interaction patterns could influence both engineered features and generated mouse-trace images. Furthermore, the impact of device type and handedness on the results remains unclear, as models trained on mouse-tracing data tend to reflect device type and handedness bias [59]. In addition, PD often affects one side of the body more than the other, and it is not certain that participants’ dominant hands were the ones most affected by the disease. Future work should include controlled data collection using standardized hardware and display settings to calibrate model performance and validate findings under more reproducible conditions.

### Conclusions

In this early-stage pilot feasibility study, we provide exploratory and hypothesis-generating evidence that models trained using weaker labels of “suspected PD” from a community-recruited sample can capture motor patterns that generalize to predicting diagnosed PD. However, due to the use of self-reported and heterogeneous labels, particularly within the suspected PD group, these results should not be interpreted as evidence of clinical screening utility and should serve as a basis for future extramurally funded work conducted using clinically confirmed cohorts in conjunction with lower-resourced community samples.

## Abbreviations

CNN: convolutional neural network
DenseNet-201: Densely Connected Convolutional Network-201
FN: false negative
FP: false positive
GradSHAP: Gradient Shapley Additive Explanations
MobileNet V2: Mobile Network v2
PD: Parkinson disease
ResNet-50: Residual Network-50
SHAP: Shapley Additive Explanations
SwinT: Shifted Window Transformer
TN: true negative
TP: true positive
ViT: Vision Transformer
